# Genomic Surveillance of SARS-CoV-2 in México: Three Years since Wuhan, China’s First Reported Case

**DOI:** 10.3390/v15112223

**Published:** 2023-11-08

**Authors:** Juan Daniel Lira-Morales, Osvaldo López-Cuevas, José Andrés Medrano-Félix, Jean Pierre González-Gómez, Irvin González-López, Nohelia Castro-Del Campo, Bruno Gomez-Gil, Cristóbal Chaidez

**Affiliations:** 1 Centro de Investigación en Alimentación y Desarrollo A.C., Coordinación Regional Culiacán, Culiacan 80110, Mexico; daniel.lira@ciad.mx (J.D.L.-M.); osvaldo.lopez@ciad.mx (O.L.-C.); jeanpierregg2@gmail.com (J.P.G.-G.); irvin.gonzalez@ciad.mx (I.G.-L.); ncastro@ciad.mx (N.C.-D.C.); 2 Investigadoras e Investigadores por México-Centro de Investigación en Alimentación y Desarrollo A.C., Coordinación Regional Culiacán, Culiacan 80110, Mexico; jose.medrano@ciad.mx; 3 Centro de Investigación en Alimentación y Desarrollo A.C., Coordinación Regional Mazatlán, Mazatlan 82112, Mexico; bruno@ciad.mx

**Keywords:** COVID-19, SARS-CoV-2, genomic surveillance, metadata

## Abstract

Objective: The aim of this work was to analyze the metadata of the SARS-CoV-2 sequences obtained from samples collected in Mexico from 2020 to 2022. Materials and Methods: Metadata of SARS-CoV-2 sequences from samples collected in Mexico up to 31 December 2022 was retrieved from GISAID and manually cured for interpretation. Results: As of December 2022, Mexican health authorities and the scientific community have sequenced up to 81,983 SARS-CoV-2 viral genomes deposited in GISAID, representing 1.1% of confirmed cases. The number of sequences obtained per state corresponded to the gross domestic product (GDP) of each state for the first (Mexico City) and the last (Tlaxcala). Approximately 25% of the sequences were obtained from CoViGen-Mex, an interdisciplinary initiative of health and scientific institutions to collect and sequence samples nationwide. The metadata showed a clear dominance of sequences retrieved by women. A similar variant distribution over time was found in Mexico and overseas, with the Omicron variant predominating. Finally, the age group with the highest representation in the sequences was adults aged 21 to 50 years, accounting for more than 50% of the total. Conclusions: Mexico presents diverse sociodemographic and economic characteristics. The COVID-19 pandemic has been and continues to be a challenge for collaboration across the country and around the world.

## 1. Introduction

SARS-CoV-2 is a single-stranded RNA virus with genome sizes of 26 to 32 kb from the coronavirus family and the etiological agent of the COVID-19 pandemic. Given its global distribution, rapid understanding of genomic features has improved our knowledge of this systemic disease. SARS-CoV-2 has caused 6.8 million deaths and more than 752 million confirmed cases, but hundreds of thousands of them would likely die from long-term COVID-19 symptoms [1]. Data from the World Health Organization shows that the most affected regions are Europe, the Western Pacific, and the Americas; Mexico has 7.3 million cases [1], and 331,365 deaths have been reported as of December 2022, showing the burden of this disease on the Mexican population. In addition, the number of deaths not confirmed by PCR but presenting symptoms is estimated to be 77.7% higher related to COVID-19, for an official tally of 505,746 [2]. The biggest challenge with COVID-19 is not to be lulled into thinking it is a seasonal flu-like illness, as variants that pose an existential threat to us are still on the rise. Therefore, it is important to build a solid genomic database of the virus and its variants. SARS-CoV-2 genomic data are available in databases such as NCBI (National Center for Biotechnology Information) and GISAID [3], both of which are crucial for in silico studies focusing on phylogenetic analysis, protein prediction, evolution and mutation analysis, etc. However, both databases rely on information from researchers worldwide; therefore, ongoing screening and sequencing support is still needed. Almost three years have passed since the first sequence was reported in a 41-year-old male patient in Wuhan, China. The sequence was reported to be 29,903 nucleotides long and was then named 2019-nCoV [4]. Since this first sequence, more than 14 million have been added to GISAID [3]. In Mexico, the first sequence of 29,849 bp from a 35-year-old patient was submitted to GISAID in March 2020 [5], with the average time from collection to report for the first 100 sequences reported in Mexico being 77 days [6]. To date, more than 80,000 sequences have been reported by multiple institutions in the country [3]. The creation of the Mexican Consortium for Genomic Surveillance (CoViGen-Mex) from the joint effort of public institutions in the country represented a milestone for genomic surveillance in Mexico, contributing more than 19,000 sequences to the total number of sequences reported in Mexico [7]. Since the start of the pandemic, genomic surveillance in Mexico has been used to identify multiple introduction events across the country and has shed light on SARS-CoV-2s dispersion routes. Additionally, when comparing the Mexican sequences to the original Wuhan strain, particularly in the spike protein, it was possible to identify the nucleotidic variation [8]. Furthermore, genomic surveillance reveals that the northern part of the country has more variants than the center and southern parts; this can be attributed to the border with the U.S. and the corresponding influx of people. However, in 2021, B.1.1.519 was the predominant variant nationwide, accounting for nearly 90% of cases [9]. With this information, by February 2022, four waves of SARS-CoV-2 cases in Mexico had ended with the nationwide predominance of the Omicron variant. At the same time, the impact of vaccination was correlated with the resurgence of COVID-19 cases in Mexico [10]. The genomic information on SARS-CoV-2 samples from Mexico has contributed to public action to mitigate the impact of COVID-19 on the Mexican population. Therefore, the retrospective analysis presented here aims to better understand and analyze the metadata of SARS-CoV-2 sequences generated in Mexico three years after the first case reported in Wuhan, China.

## 2. Materials and Methods

A total of 81,983 submission metadata points were used in this study. Only sequences from samples collected in Mexico up to 31 December 2022 and deposited in the GISAID database (https://www.gisaid.org/) were included in this retrospective analysis. The metadata was extracted via the download tool in the EpiCoV section with the custom filter “Mexico” in the country menu (Table S1). All information is available at https://doi.org/10.55876/gis8.230130ua (accessed on 23 January 2023). Manual curation was performed to standardize the name and language in which the departments (states), gender, and submitting laboratory were reported. Greek alphabet labels have been added to the most representative VOCs (Variants of Concern) in Mexico. The remainder were classified as “other.” The figures were generated using RStudio v2022.12.0 Build 353 [11]. Additional data on confirmed cases and the total state population were obtained from https://datos.covid-19.conacyt.mx/ (accessed on 26 January 2023).

## 3. Results

**Figure 1:**
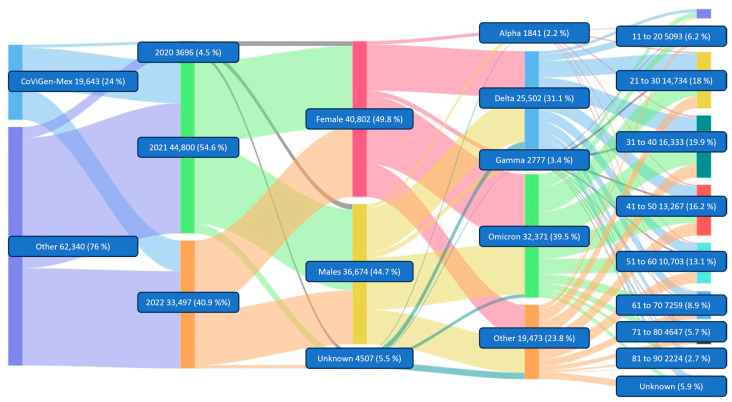
Metadata distribution of SARS-CoV-2 sequences from samples collected in Mexico and categorized by institution of origin, year of submission, sex, SARS-CoV-2 variant, and age group.

Mexico is among the top twenty countries whose populations have suffered the most from the COVID-19 pandemic. It also ranks fifth with the most devastating dice effect [1]. As of December, 81,983 SARS-CoV-2 sequences collected in Mexico were registered in the GISAID database as part of the genomic surveillance of new variants. Of the total sequences, around 25% were submitted by CoViGen-Mex and thus contributed significantly to the genomic monitoring of SARS-CoV-2 (Figure 1).

However, the joint efforts of all institutions only succeeded in sequencing around 1.1% of all confirmed cases. It is important to note that statistical estimates suggest that sequencing 5% of reported cases is necessary to detect emerging variants when they occur at intervals of 0.1 and 1% [12].

### 3.1. Geographical Sequences Representation

**Figure 2:**
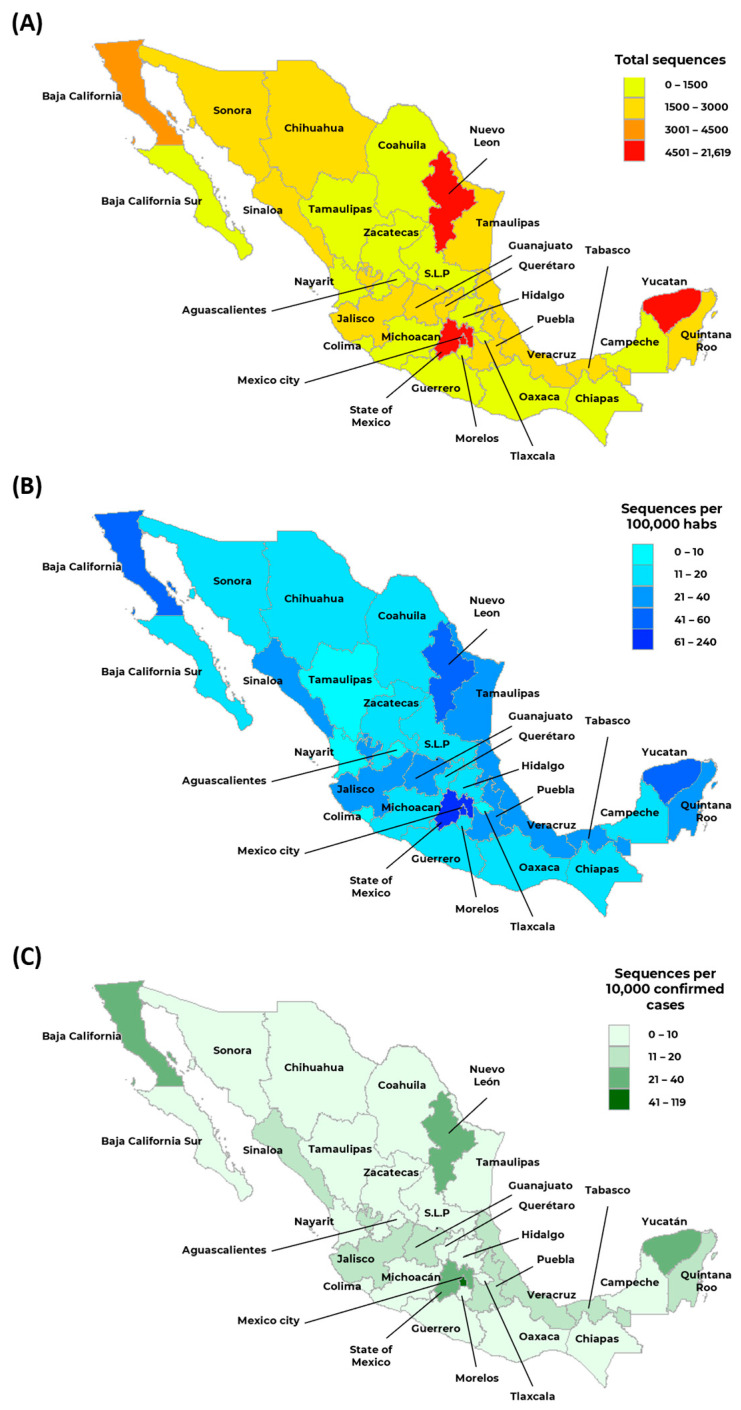
Distribution of sequences per state in Mexico. (**A**) Sequences reported per state (Collected from February 2020 to December 2022); (**B**) Sequences reported per 100,000 inhabitants; and (**C**) Sequences reported per 10,000 confirmed cases.

The raw number of sequences obtained from the samples collected per state of the Mexican Republic shows a large discrepancy between states (Figure 2). For example, the state with fewer sequences was Tlaxcala with 0.34%; however, Mexico City has more than 26% of the total sequences.

For perspective, the five states with more sequences (Mexico City, the State of Mexico, Yucatan, Nuevo Leon, and Baja California) represent 52% of the country’s total. Meanwhile, the last five states (Guerrero, Colima, Durango, Nayarit, and Tlaxcala) account for only 3.3% of the total, showing differences between states in the number of sequences reported to GISAID. To better understand these numbers proportional to population with the representation of sequences per 100,000 inhabitants, the same states remain at the top: Mexico City, with 240 sequences per 100,000 inhabitants, and below, Tlaxcala, with only three sequences per 100,000 inhabitants, with an average value per state of 28 sequences per 100,000 inhabitants. Analysis of the number of sequences per 10,000 confirmed cases shows the same pattern of representation of the number of sequences per state, with Tlaxcala having only two sequences per 10,000 confirmed cases and Mexico City having 119 sequences per 10,000 confirmed cases.

### 3.2. Sequence by Institution of Origin in Mexico

The analysis of the sequences collected in Mexico between February 2020 and December 2022 was divided into two groups: the Mexican Consortium for Genomic Surveillance (CoViGen-Mex), a nationwide initiative of institutions, and the other group made up of institutions not part of CoViGen-Mex, such as individual efforts from universities, research centers, and private laboratories across the country. The former is due to the importance of creating a surveillance system to combat SARS-CoV-2, which represents a milestone in the country for the joint response of specialists and infrastructure. The CoViGen-Mex in 2022 was responsible for almost 25% (19,643) of the total number of sequences in the country and contributed to the pandemic with 4.5% of the total as of 2020, while 2021 was the year with 54.6%. The total number of sequences reported by CoViGen-Mex to date has fallen to just 40.9% of the total by 2022—the same percentage representation of all other institutions’ sequences per year. In 2020, 3686 sequences were retrieved nationwide in Mexico, increasing to 44,800 in 2021, the year with more sequences in the country (54.6%). In 2022, 33,497 sequences were deposited in GISAID, a lower number than in 2021.

### 3.3. Data by Sex and Age

**Figure 3:**
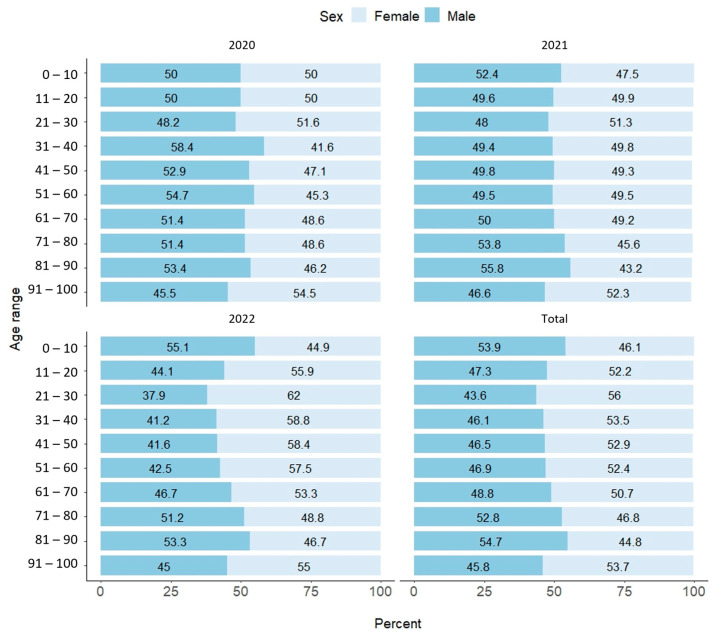
Sex distribution of sequences collected from Mexico annually Metadata with unknown information is not shown. In all cases, the percent values were rounded to two decimals.

Data retrieved from GISAID were classified into three categories: male, female, and other if the submitter did not provide information. This last category accounts for 5.5% of the total. In Mexico, sequences recovered from SARS-CoV-2 cases were female, with 49.8% of all cases being female; 44.7% were men. This behavior has not changed significantly for the Mexican population. In the first year of the pandemic [13], 42.4% of SARS-CoV-2 sequences came from women and 46.8% from men, with no information available for 10.8% of patients. The number of cases in men and women in Mexico remains the same in both sexes (46.8% and 53.1%) and remains proportional to the sequences retrieved. However, this is not always the case worldwide. For example, in Singapore, more than 90% of confirmed cases were male. In contrast, in Ukraine, only 40% of cases affected men [14]. The age group most affected by SARS-CoV-2 was recorded between 31 and 40 years (19.9%), closely followed by the groups of 21 to 30 years (18%) and 41 to 50 years (16.2%). Interestingly, some cases were reported in newborns, in contrast to a few other cases (0.5%) in other individuals (Figure 3).

Looking at the general distribution in most data ranges in 2020–2022, more sequences were retrieved from samples from women (*n* = 81,983), although in the first year of 2020, the opposite was true when sequences collected by men predominated. However, fewer sequences were retrieved this year (*n* = 3683). Interestingly, the data shows a balanced representation of both genders until 2021, the year with more sequences (*n* = 44,800), and finally the data trend towards a more female representation of sequences until 2022 (*n* = 33,497).

### 3.4. SARS-CoV-2 Variants in Mexico

**Figure 4:**
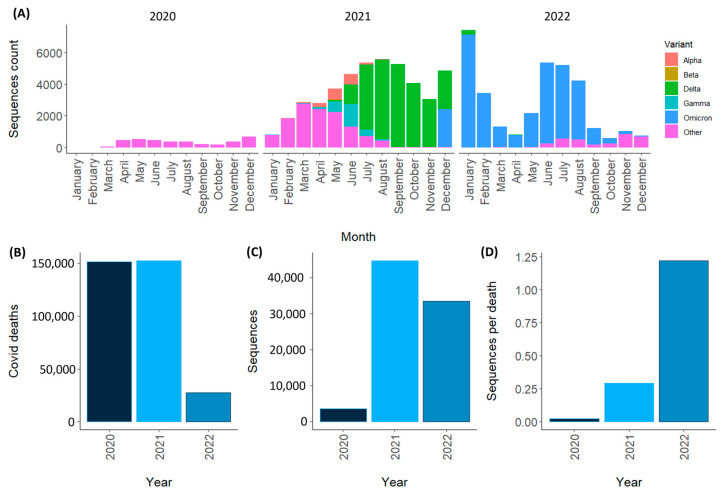
SARS-CoV-2 presence in Mexico, 2020–2022. (**A**) Variant distribution of SARS-CoV-2 on samples collected in Mexico in 2020–2022. (**B**) Deaths associated with COVID-19 in Mexico per year. (**C**) Sequences reported from samples taken in Mexico per year. (**D**) Number of sequences per defunction in Mexico per year.

As for variants of concern (VOCs) in Mexico over time, the Alpha variant (B.1.1.7; first reported: September 2020, United Kingdom; VOC: 12.18.2020) was first detected in December 2020, with notable presence to date August 2021 (*n* = 1841). The beta variant (B.1.351; first reported: May 2020, South Africa; VOC: 12 August 2020) was the least found variant (*n* = 19) of all VOCs in samples from Mexico. The gamma variant (P.1; first reported: November 2020; VOC: 1 November 2021) was first discovered in Mexico in January 2021, was the most common variant until August 2021 (*n* = 2777), and, interestingly, existed alongside the alpha variant punctual. The Delta variant (B.1.617.2: first reported: October 2020, India; VOC: 5 November 2021) was first detected in Mexico in early 2021, with higher numbers between July and December 2021 (*n* = 25,502). be replaced by the Omicron variant (B.1.1.529; first report: November 2021; VOC: 26 November 2021) with representation until December 2022 (*n* = 32,371) (Figure 4). The remaining sequences retrieved (*n* = 19,473) were not from VOCs reported by the OMS [1].

Globally, a similar distribution of variants was observed over time, with the predominance of the Alpha variant (April 2021–June 2021), the Delta variant becoming the predominant one (July 2021–December 2021), and finally, this being the case, the complete predominance of the Omicron variant from January 2022 to today [15]. According to the SISVER (National Epidemiological Surveillance System for Respiratory Diseases), a total of 331,560 deaths were caused by COVID-19 in Mexico from 2020 to 2022. Regarding the number of sequences submitted to GISAID in the first year of the pandemic, only 3686 were submitted, which corresponds to a coefficient of 0.025 sequences for each reported death related to COVID-19. In contrast, the coefficient would be 1.22 by 2022. This number can be interpreted in both directions, considering that there were the largest number of deaths at the beginning of the pandemic, and in 2022, fewer people died from COVID-19, but more sequences were submitted. This is due to the research groups’ experiences during the pandemic [2].

Data from the national COVID-19 dashboard provided a complete nationwide record of daily COVID-19 cases, clearly showing the impact and patterns of the five waves of COVID-19 in Mexico. Of the first confirmed cases on 27 February 2020, the maximum total is 417,321 cases for the first wave in July 2020, 1,687,718 cases for the second wave in January 2021, 3,120,927 cases for the third wave during the fourth wave in August 2021, and 4,943,564 cases for the fourth wave in January 2022, with the highest number of daily cases at 75,305 and 6,474,405 cases for the fifth wave in July 2022 [16]. In this regard, those with COVID-19 were in Mexico associated with comorbidities such as hypertension (11.9%), obesity (9.59%), diabetes (8.74%), and tobacco use (5.41%). Given the severity of COVID-19, many people had to be hospitalized. In this context, Rojo-del Moral 2022 reports 116,644 patients, 45% of whom were hospitalized at the Mexican Social Security Institute (IMSS), and a mortality rate of about 43%. High blood pressure and diabetes are the most common comorbidities [17].

## 4. Discussion

Metadata analysis presents technical challenges worldwide, from sample collection to the number of days elapsed to the sequencing and reporting of results. In addition, to facilitate their study, metadata standardization of the information is still required [18], as well as to prevent spelling errors and inconsistent naming conventions [6,19], in order to obtain valuable data for researchers and authorities. In connection with the latter, and to better understand the entire metadata generation process, more data science specialists need to integrate all the data generated during the Omic era.

Globally, only 6.8% of countries have sequencing levels of 5% or higher. Meanwhile, 45.5% have sequencing values per case of 0.5% or less [20]. These data confirm the need for further genomic surveillance efforts by all participating institutions across the country. This variability has significant implications for global phylogeographic analysis of SARS-CoV-2 variants [6]. Different countries reported different percentages of whole genome sequencing. However, compared to documented COVID-19 episodes, this is relatively small due to the availability of economic, technological, and human experts to generate data. Consequently, genomic surveillance is carried out by a limited number of laboratories [21].

Globally, there are notorious discrepancies between low- and high-income countries, with an average of 11 sequences per 1 million population in low-income countries; there are now an average of more than 5000 sequences per 1 million inhabitants in high-income countries [22]. In this study, there is a correlation between the Gross Domestic Product (GDP) for the first and last states in all categories of representation of the sequence, meaning that the GDP of Tlaxcala represents 0.6% of the entire country; on the other hand, the GDP of Mexico City is 15.8% [23].

There is no homogeneous behavior, which may be due to the different availability of facilities per state for whole genome sequencing but also to the different number and spatio-temporal increase of COVID-19 in each state [13]. This information shows a clear difference between the sequences retrieved by the state. To better understand this phenomenon, the data must be more representative of the overall population to facilitate genomic surveillance of this pandemic and future massive transmission events. These hardest-hit states nationwide belong to heavily touristic areas and have high visitor fluctuations. Therefore, strict measures have been taken to detect new variants and increase the likelihood of new variants being introduced [13]. Travel restrictions play an essential role in reducing the transmission of SARS-CoV-2, and this measure helps slow the spread of the virus as a preventative strategy [24,25]. The COVID-19 pandemic has shown anachronistic behavior in all Mexican states. The spatio-temporal reporting cases are not homogeneous, as in most countries worldwide, and are consistent with the spatio-temporal dispersion of zoonotic diseases [26]. At the same time, the number of sequences received by states and their proportion per 100,000 population fluctuates due to adjustments and the establishment of laboratories by research institutions or state governments for SARS-CoV-2 determinations in 2020 and early 2021.

Due to the high burden of disease in Mexico, health authorities have implemented the CoViGen-Mex initiative (the Mexican Consortium for Genomic Surveillance), carried out within the framework of the National Virology Research and Incidence Project of CONAHCyT (National Council for Humanity, Sciences, and Technologies), in collaboration with the Institute of Epidemiological Diagnosis and Reference (InDRE), according to the guidelines of the Mexican Health Secretariat. To obtain more SARS-CoV-2 sequences to identify variants circulating in Mexico and determine its demographic behavior, member enrollment in CoViGen-Mex is dynamic. So far it consists of the Mexican Institute of Social Security, the National Institute of Respiratory Diseases of Mexico (INER), the National Autonomous University of Mexico (UNAM), the Center for Research and Advanced Studies of the National Polytechnic Institute (CINVESTAV) and the Research Center for Nutrition and Development (CIAD), Autonomous University of Mexico City (UACM), Autonomous University of the State of Morelos, National Institute of Cardiology, Autonomous University of San Luis Potosí (UASLP), Metropolitan Autonomous University (UAM), National Institute of Genomic Medicine (INMEGEN), National Institute of Public Health of Mexico (INSP), University of Oxford and the Free University of Berlin (FU Berlin).

The creation of a multidisciplinary consortium is not a disadvantage for Mexico globally. Similar organizations were founded to combat the SARS-CoV-2 pandemic and bring together research institutes, academies, and government institutions. One example is the COVID-19 Genomics UK (COG-UK) consortium, which aims to integrate sequencing data and metadata from the United Kingdom [25]. In India, the INSACOG (Indian SARS-CoV-2 Genomics Consortium) sequences 5% of all positive cases nationwide [27]. In all cases, collaborative efforts across the country or worldwide, such as the Coronavirus Immunotherapeutic Consortium, are required to analyze the therapeutic targets of SARS-CoV-2 [28].

Taboada et al. (2021) found that the Mexican population has a median age of 44 years (with an interquartile of 32–58 years, with a minimum of less than one year and a maximum of 105 years) [10]. Kammar-García et al. (2020) [29] found that comorbidities such as obesity and diabetes may be associated with a younger age of severe disease in the Mexican population. It is important to consider that although mortality rates are low in younger people, rates of severe and critical infections are higher, with unknown future impacts on the population [30].

All variants of concern (VOCs) reported by the OMS have been detected in Mexico. At the end of this period, the Omicron variant replaced all others with almost 40% of the total reported sequences, followed by the Delta variant with 31.1% of all other variants. Although this lineage has been reported primarily in Mexico and the USA, it has also been reported in Canada, South America, and European countries [13]. This lineage evolved from the B.1.1.222 lineage that was previously dominant in Mexico [31]. The Omicron lineage has been described worldwide as the most mutated variant and with the highest transmissibility by evading the immune system [32,33]. Cedro-Tanda et al. (2022) observed an increase in the prevalence of the Omicron variant in Mexico City by up to 88% from 16 November to 31 December 2021 [34]. This lineage has prevailed, but its effects have been less devastating, perhaps due to vaccination [35,36]. The CoViGen-Mex database makes its complete SARS-CoV-2 genome sequences available in the Global Initiative on Sharing All Influenza Data (GISAID) through its EpiCoV section, which together with NCBIs GenBank are the primary repositories for SARS-CoV 2 genome sequences. Although CoViGen-Mex was established in February 2021, through this program, Mexico City reported SARS-CoV-2 sequences obtained from samples previously stored since the start of the COVID-19 pandemic. Although sequencing of clinical samples is difficult to implement and limited to the number and distribution of samples available, significant efforts have been made in this regard. It is also important to note that the epidemiology of SARS-CoV-2 can be strengthened through alternatives such as wastewater-based epidemiology (WBE). In Mexico, WBE efforts have been made, with some cases seeing spikes in SARS-CoV-2 detection in water almost two weeks before clinical cases re-emerged [37]. Similar nationwide studies have demonstrated the correlation between positive water samples and clinical cases, primarily from wastewater treatment plant influent samples [38]. Even more so, in the southern region of Mexico, methods for detecting SARS-CoV-2 in water have been developed to detect about ten new COVID-19 cases per day [39]. As can be seen, further efforts are needed to utilize alternatives to clinical sequencing. This study is limited by the number of samples deposited in GISAID. In addition, it is not within the scope of this study to determine the sampling method of each supplier or to determine the bias in the number of samples submitted by healthcare facilities and laboratories in each region. Further efforts are needed nationwide to homogenize the number of samples and the availability of infrastructure for all institutions involved in genomic surveillance.

COVID-19 caused profound stress on the Earth’s population. It occurred at the end of 2019. Travelers helped spread the virus worldwide, and we are experiencing a global event very quickly. More people have died from this coronavirus strain in the past three years than in previous years. Our results show the panorama of SARS-CoV-2 variants circulating in Mexico from 2020 to 2022. Furthermore, genomic surveillance is the best tool to demonstrate the spread of specific SARS-CoV-2 lineages in Mexico. All viruses change their genomic rearrangements with transmission frequency. These changes could affect the ability and speed of the virus to spread, the severity of the disease, the effectiveness of the vaccine, diagnostic methods, and containment strategies. Therefore, genomic surveillance in Mexico can serve various purposes. (1) genomic legacy of SARS-CoV-2; (2) early detection and tracking of new variants; (3) introduction/dissemination of non-Mexican variants; (4) future studies on evolutionary viral genomics; (5) a valuable genomic database for vaccine development; (6) other initiatives such as Maintain and develop CoViGen-Mex with sufficient funding to maintain genomic surveillance not only for SARS-CoV-2 but also for other microorganisms of public health importance, and finally, (7) promote training courses in data science at universities to train highly specialized professionals who deal with the ever-growing data from genomic analyses.

## Data Availability

All data used in this study are publicly available through GISAID database.

## Supplementary Materials

The following supporting information can be downloaded at: https://www.mdpi.com/article/10.3390/v15112223/s1, Table S1: GISAID supplemental table.
