# Supplementary material for: Genomic Surveillance of SARS-CoV-2 in México: Three Years since Wuhan, China’s First Reported Case
Source: Viruses. 2023 Nov 8;15(11):2223. doi: 10.3390/v15112223 (PMC10674944; doi:10.3390/v15112223)
Supplement: Supplementary file 1 [file viruses-15-02223-s001.zip › viruses-2701866-Table S1.pdf]

SUPPLEMENTAL TABLE

Data Availability

GISAID Identifier:	EPI_SET_230130ua
doi:	10.55876/gis8.230130ua

All genome sequences and associated metadata in this dataset are published in GISAID's EpiCoV database. To view the contributors of each individual sequence with details such as accession number, Virus name, Collection date, Originating Lab and Submitting Lab and the list of Authors, visit 10.55876/gis8.230130ua

Data Snapshot

- EPI_SET_230130ua is composed of 81,983 individual genome sequences.
- The collection dates range from 2020-02-27 to 2022-12-26;
- Data were collected in 1 countries and territories;
- All sequences in this dataset are compared relative to hCoV-19/Wuhan/WIV04/2019 (WIV04), the official reference sequence employed by GISAID (EPI_ISL_402124). Learn more at https://gisaid.org/WIV04.
